# Noninvasive continuous optical monitoring of absolute cerebral blood flow in critically ill adults

**DOI:** 10.1117/1.NPh.5.4.045006

**Published:** 2018-11-23

**Authors:** Lian He, Wesley B. Baker, Daniel Milej, Venkaiah C. Kavuri, Rickson C. Mesquita, David R. Busch, Kenneth Abramson, Jane Y. Jiang, Mamadou Diop, Keith St. Lawrence, Olivia Amendolia, Francis Quattrone, Ramani Balu, W. Andrew Kofke, Arjun G. Yodh

**Affiliations:** a University of Pennsylvania, Department of Physics and Astronomy, Philadelphia, Pennsylvania, United States; b University of Pennsylvania, Department of Anesthesiology and Critical Care, Perelman School of Medicine, Philadelphia, Pennsylvania, United States; c Western University, Department of Medical Biophysics, London, Ontario, Canada; d Lawson Health Research Institute, Imaging Division, London, Ontario, Canada; e University of Campinas, Institute of Physics, Campinas, São Paulo, Brazil; f University of Texas Southwestern, Department of Neurology and Neurotherapeutics, Dallas, Texas, United States; g University of Texas Southwestern, Department of Anesthesiology and Pain Management, Dallas, Texas, United States; h University of Pennsylvania, Department of Neurosurgery, Perelman School of Medicine, Philadelphia, Pennsylvania, United States; i University of Pennsylvania, Department of Neurology, Perelman School of Medicine, Philadelphia, Pennsylvania, United States

**Keywords:** diffuse correlation spectroscopy, time-resolved contrast-enhanced near-infrared spectroscopy, absolute cerebral blood flow, brain-injured patients, continuous monitoring

## Abstract

We investigate a scheme for noninvasive continuous monitoring of absolute cerebral blood flow (CBF) in adult human patients based on a combination of time-resolved dynamic contrast-enhanced near-infrared spectroscopy (DCE-NIRS) and diffuse correlation spectroscopy (DCS) with semi-infinite head model of photon propogation. Continuous CBF is obtained via calibration of the DCS blood flow index (BFI) with absolute CBF obtained by intermittent intravenous injections of the optical contrast agent indocyanine green. A calibration coefficient (γ) for the CBF is thus determined, permitting conversion of DCS BFI to absolute blood flow units at all other times. A study of patients with acute brain injury (N=7) is carried out to ascertain the stability of γ. The patient-averaged DCS calibration coefficient across multiple monitoring days and multiple patients was determined, and good agreement between the two calibration coefficients measured at different times during single monitoring days was found. The patient-averaged calibration coefficient of $1.24\times 10^{9}\;(\mathrm{mL}/100\;g/\min)/({\mathrm{cm}}^{2}/s)$ was applied to previously measured DCS BFI from similar brain-injured patients; in this case, absolute CBF was underestimated compared with XeCT, an effect we show is primarily due to use of semi-infinite homogeneous models of the head.

## 1 Introduction

Brain-injured patients are prone to development of secondary brain injury during the management period, and therapeutic interventions for secondary brain injury hold potential for meaningful impact on long-term patient outcome.^1^ More specifically, clinical studies suggest that brain-injured patients with impaired cerebral autoregulation are unusually susceptible to secondary ischemic insult^2^^,^^3^ and that when cerebral blood flow (CBF) drops below a critical threshold, the development of irreversible tissue damage can occur;^4^ whereas if CBF exceeds the brain’s metabolic requirement, unregulated hyperemia can cause brain edema.^5^^–^^7^ Thus, CBF monitoring during the management period can improve acute care via rapid detection and resultant therapy to attenuate conditions that can lead to secondary brain injury.^1^

To date, several measurement methods, including PET,^8^ MRI,^9^ ${\mathrm{Xe}}^{133}$,^10^ Xe-CT,^11^^,^^12^ transcranial Doppler,^13^^,^^14^ thermal diffusion,^15^ jugular bulb oximetry,^16^ and EEG^17^^,^^18^ have been utilized to infer or to directly evaluate CBF, but none of these offers truly continuous noninvasive absolute CBF monitoring capabilities at the bedside. The present study explores the potential of a combination of near-infrared (NIR) optical methods for noninvasive continuous bedside monitoring of absolute CBF in a cohort of adult human patients with brain injury. Thus, patient treatment needs not be interrupted, and risks of secondary insults from patient transport (e.g., to MRI, PET instruments) are reduced. The approach uses time-resolved dynamic contrast-enhanced near-infrared spectroscopy (DCE-NIRS) to calibrate the blood flow index (BFI) determined by diffuse correlation spectroscopy (DCS); similar brain volumes are sampled by the two techniques, and the same light transport model is used for data analysis and generation of DCS calibration coefficients. The calibrated BFI subsequently permits continuous bedside monitoring of CBF in physiological units, e.g., mL of blood/100  g/min.^19^^,^^20^ In this contribution, to establish the repeatability of the calibration coefficients, we cross calibrate these two quantitatively different and complementary NIR optical blood flow (BF) measurement techniques at different time points during the care of a cohort of patients with acute brain injury. We also determine an average calibration coefficient based on data across multiple monitoring days and multiple patients. Finally, we apply this calibration coefficient to assess flow in critically ill patients with subarachnoid hemorrhage from a previous study carried out in our groups.^21^

DCS employs NIR light to directly and noninvasively measure local microvascular CBF.^22^^–^^26^ This optical technique is suitable for continuous bedside monitoring; it has excellent temporal resolution,^27^ offers penetration into cerebral tissues through intact scalp and skull,^28^ and is compatible for combined use with traditional NIRS devices.^21^^,^^25^^,^^29^^–^^31^ To date, DCS-measured relative BF changes have been extensively validated in many tissues and against numerous other techniques,^21^^,^^23^ but the DCS BFI has unusual units (${\mathrm{cm}}^{2}/s$). Absolute calibration of DCS in traditional units (mL/100  g/min) would enable other useful CBF assessments of the physiological condition of brain-injured patients, e.g., at baseline and via patient comparison. Recently, a DCE-NIRS method that employs injection of a light-absorbing optical contrast agent, indocyanine green (ICG), was demonstrated to measure absolute CBF.^19^^,^^32^^–^^34^ Specifically, the time-dependent absorption signal of an ICG bolus through cerebral tissue enables measurement of absolute CBF at a single time point. This approach was applied to measure CBF in piglets and adult pigs,^20^^,^^32^^,^^35^ newborn infants,^36^^,^^37^ and healthy adults.^34^^,^^38^^,^^39^ Notably, in a piglet study, comparisons of DCE-NIRS and CT perfusion measurements showed good agreement.^32^ Unfortunately, absolute CBF measurement by DCE-NIRS requires injection of an ICG bolus and is also limited by a maximum recommended daily dose; both factors are barriers to continuous CBF monitoring.

Here, we investigate a scheme for noninvasive continuous bedside monitoring of absolute CBF in adult human brain-injured patients based on a combination of time-resolved DCE-NIRS and DCS. By comparing DCE-NIRS to concurrent DCS measurements, a calibration coefficient for the CBF is determined that permits conversion of DCS BFI into absolute BF units at other times during injury management. The concurrent measurements enabled assessment of the stability of ICG calibration coefficients obtained from each patient across single monitoring days. We found excellent agreement between the two calibration coefficients across single monitoring days. Additionally, we determined a patient-averaged calibration coefficient for DCS monitoring of absolute CBF. Significant correlation between CBF obtained with two optical techniques was also observed. Finally, the population-averaged calibration coefficient was applied to a group of adult brain-injured patients studied previously by our group,^21^ thereby taking first steps toward using DCS directly as a measure of absolute CBF. In total, the research suggests that the combined DCS and DCE-NIRS method should enable improved continuous, noninvasive, and quantitative DCS monitoring of absolute CBF at the bedside in the clinic.

## 2 Methods

### 2.1 Dynamic Contrast-Enhanced Time-Resolved Near-Infrared Spectroscopy

The DCE-NIRS technique treats the brain vascular bed as a linear, time-invariant system with single entrance and exit. CBF can be measured using DCE-NIRS by tracking the concentration of a NIR tracer in the brain vascular bed.^32^^,^^40^^,^^41^ Specifically, when a bolus of ICG-tracer is injected intravenously in a peripheral or central vein, then the rate of delivery of ICG to the brain vascular bed at time t is $\mathrm{CBF}\cdot C_{a}(t)$, where $C_{a}(t)$ [$\mu \mathrm{mol}\cdot L^{-1}$] is the cerebral arterial ICG concentration (per volume of blood). If the amount of ICG in brain tissue varies linearly with respect to the arrival rate, and if CBF is constant in time (i.e., during the measurement), then the accumulated ICG concentration (per volume of tissue) in the brain tissue at time t, i.e., Q(t) [$\mu \mathrm{mol}\cdot L^{-1}$] is^32^^,^^33^

$$Q(t)=\mathrm{CBF}\cdot C_{a}(t)*R(t),$$ (1)

where * is the convolution operator, and R(t) is the so-called impulse residue function, which can be viewed as the fraction of ICG remaining in cerebral vascular bed at time t following an instantaneous ICG bolus into the arterial input at time zero, i.e., $C_{a,0}\delta (t)$. Deconvolution with respect to Q(t) and $C_{a}(t)$ permits CBF·R(t) to be extracted.^33^ The magnitude of CBF·R(t) at the initial time gives absolute CBF [mL/100  g/min] as R(0)  =1. (Note, a cerebral tissue density of 1.05  g/mL was used to convert the CBF units of mL/100  mL/min obtained from the deconvolution to mL/100  g/min.)

**Fig. 1:**
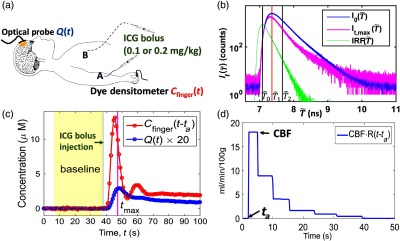
(a) DCE-NIRS measurement of absolute CBF utilizes TR-NIRS to track the passage of an ICG bolus [rapidly injected (<2  s) in a peripheral (location A) or central vein (location B)] through the tissue sampled by the optical probe. The average ICG concentration across the tissue volume, i.e., Q(t), is measured by TR-NIRS (Sec. 2.1.1). The cerebral arterial ICG concentration is approximated using the arterial ICG concentration in the finger, i.e., $C_{\text{finger}}(t)$, which is measured with a customized dye densitometer (Sec. 6 and Appendix 1). (b) Exemplar effects on the TR-NIRS signals from a 0.2  mg/kg IV ICG bolus injection. Note, the TR-NIRS measurement is a histogram of the number of photons striking the detector as a function of the time difference, $\tilde{T}$, between the TR-NIRS source pulse trigger and the detected photons. The TR-NIRS measurement acquired at the time of maximal ICG concentration in the brain [i.e., $I_{t,\max}(\overset{˜}{T})]$ is attenuated relative to the baseline TR-NIRS measurement acquired prior to ICG injection [i.e., $\;I_{0}(\tilde{T})$]. Here, $\overset{˜}{T_{0}}$ denotes the maximal photon count bin of the IRF, and $\overset{˜}{T_{1}}$ and $\overset{˜}{T_{2}}$ denote the histogram bins with maximal and half-maximal photon counts in the $I_{0}(\tilde{T})$ curve. (c) Temporal measurements of Q(t) [via Eq. (2)] and $C_{\text{finger}}(t)$ in the same exemplar patient. The peak value of Q(t) corresponds to an $0.065\;{\mathrm{cm}}^{-1}$ absorption change due to ICG. (d) The CBF-scaled impulse residue function, R(t), retrieved from the deconvolution of the curves in (c). The height of the initial plateau yields CBF (18  mL/min/100  g). Here, $t_{a}$ (2 s) is the arterial ICG bolus transit time delay between the finger arteries and the cerebral arteries, which is a fit parameter in the deconvolution.

We used time-resolved NIRS (TR-NIRS) to measure Q(t) on the forehead superior to the frontal sinuses [Fig. 1(a) and Sec. 2.1.1]. The width of Q(t) is a metric of how quickly the ICG bolus passes through the TR-NIRS sampled tissue volume [Fig. 1(c)]. Note, a previous study demonstrated high correlation (slope not significantly different from unity) between $C_{a}(t)$ measured noninvasively with a dye densitometer attached to the finger and measured invasively through the radial artery.^42^ Assuming that the ICG concentration in the radial artery and the cerebral arteries have the same temporal shape $C_{a}(t)$ is approximated by the arterial ICG concentration in the finger, i.e., $C_{\text{finger}}(t)$ measured with a custom dye densitometer [Fig. 1(c), Sec. 6 Appendix 1]. The injected ICG bolus arrives at the cerebral arteries earlier than it arrives in the finger arteries. Therefore, the arterial ICG bolus transit time-delay between the finger arteries and the cerebral arteries, i.e., $t_{a}$ is a fitting parameter in the deconvolution of Eq. (1), and the experimentally derived deconvolution [e.g., Fig. 1(d)] is $\mathrm{CBF}\cdot R(t-t_{a})$. The shape of $C_{a}(t)$ was assumed to be similar to the shape of $C_{\text{finger}}(t)$, i.e., $C_{a}(t)=C_{\text{finger}}(t-t_{a})$.

#### 2.1.1 Tissue ICG concentration measurement with TR-NIRS

TR-NIRS delivers short NIR light pulses (<100  ps) to a source position on the scalp; the pulse broadens as it propagates through tissue. At the detector, located a distance r=3.2  cm away from the source on the scalp surface, the so-called temporal point spread function (TPSF) is measured, which is a histogram of the number of photons striking the detector as a function of the time difference, $\tilde{T}$, between the TR-NIRS source pulse trigger and the detection of photons [Fig. 1(b), histogram bin width 1 ps]. To track the kinetics of ICG passage through the sampled tissue volume following intravenous bolus injection, TPSFs were continuously acquired at a single wavelength (808 nm) at a sampling rate of 0.9 Hz (i.e., histogram integration time of 1.1 s). Note that the time-domain photon diffusion equation models the distribution of photons as a function of photon time-of-flight through the tissue, i.e., ϕ(T), which is not identical to the experimentally measured TPSF because of instrument response function (IRF) effects. Using a semi-infinite homogeneous tissue model, the temporal cerebral tissue absorption changes caused by the transit of ICG through the brain can be determined via^43^

$$\Delta \mu_{a}(t)=-\frac{2}{c(T_{2}^{2}-T_{1}^{2})}\overset{T_{2}}{\underset{T_{1}}{\int }}\ln\;\frac{I_{t}(T)}{I_{0}(T)}dT.$$ (2)

Here $T\approx \overset{˜}{T}-\overset{˜}{T_{0}}$, where $\tilde{T_{0}}$ is the maximal photon count bin of the IRF measurement [Fig. 1(b), Sec. 2.3.2], $I_{0}(T)$ is the mean TPSF acquired across the 30-s interval immediately prior to injection of the ICG bolus [Fig. 1(b)], $I_{t}(T)$ is the TPSF measured at time t following ICG bolus injection, c is the speed of light in tissue, and the limits $T_{1}$ and $T_{2}$ can be chosen to bound any time interval within the TPSF. In our study, $T_{1}$ was chosen to be the time-of-flight with maximal photon count at baseline, i.e., $I_{0}(T_{1})=\max(I_{0})$, and $T_{2}$ was the time for the baseline TPSF to fall to half the maximal photon count, i.e., $I_{0}(T_{2})/I_{0}(T_{1})=0.5$; $T_{2}>T_{1}$. $T_{1}$ and $T_{2}$ were chosen to roughly balance between increased brain sensitivity (long time-of-flights) and adequate signal-to-noise ratio (very long time-of-flights have poor signal-to-noise ratio). Note, long times-of-flight are also more susceptible to fluorescence light.^44^ For $T_{1}<T<T_{2}$, Eq. (2) makes the approximation that $\ln[I_{t}(T)/I_{0}(T)]\approx \ln[\phi_{t}(T)/\phi_{0}(T)]$, where $\phi_{t}(T)$ and $\phi_{0}(T)$ denote the semi-infinite photon diffusion model computations of the photon distributions given the tissue absorption and scattering optical properties at time t and baseline, respectively. Note, according to our computer simulations with varied IRF temporal width, about 10% underestimation in CBF should be expected when using Eq. (2) to analyze experimentally measured TPSF, i.e., compared with an ideal TPSF with no broadening of the laser pulse due to electro-optical components. Moreover, we found that the CBF calculation is not very sensitive to the width of IRF [using Eq. (2)], i.e., <5% difference between IRF full-width at half-maximum (FWHM)=180 versus 550 ps.

The ICG concentration in brain tissue is then calculated from the Beer–Lambert law, i.e.,

$$Q(t)=\Delta \mu_{a}(t)/[\ln(10)\times \epsilon_{\mathrm{ICG}}],$$ (3)

where $\epsilon_{\mathrm{ICG}}$ is the spectral extinction coefficient of ICG in plasma measured at 808 nm and 6.5  μM concentration (191  OD/mM/cm^45^), and $\Delta \mu_{a}(t)$ is obtained via Eq. (2). Note that Eq. (3) assumes that changes in tissue absorption from chromophores other than ICG are negligible.

### 2.2 Diffuse Correlation Spectroscopy

DCS delivers highly coherent NIR light to a source position on the scalp, which then propagates diffusively inside the tissue. At a distance r from the source, measured along the tissue surface, the rapid speckle intensity fluctuations of the multiply scattered light are measured.^22^^,^^23^^,^^46^^–^^52^ Specifically, these fluctuations, which are mainly induced by red blood cell motion, are quantified by the normalized intensity autocorrelation function, $g_{2}(r,\tau )\equiv \langle I(r,t)I(r,t+\tau )\rangle /{\left\langle I(r,t)\right\rangle }^{2}$, where I(r,t) is the detected light intensity at time t and distance r from the source, τ is the delay-time, and the angle brackets, ⟨ ⟩, represent time-averages. The intensity autocorrelation function is related to the electric field autocorrelation function, $G_{1}(r,\tau )\equiv \langle E^{*}(r,t)\cdot E(r,t)\rangle$, by the Siegert relation, $g_{2}(r,\tau )=1+\beta {\left|G_{1}(r,\tau )/G_{1}(r,0)\right|}^{2}$.^46^^,^^47^ $G_{1}(r,\tau )$ satisfies the correlation diffusion equation in highly scattering media, and β is a constant that is inversely proportional to the number of speckles detected.

The tissue BFI and the parameter β are extracted from the fit of the measured $g_{2}(\tau )$ to the homogeneous continuous-wave solution of the correlation diffusion equation in the semi-infinite geometry via the Siegert relation. (Limitations of the semi-infinite model are explored in Sec. 4.) The semi-infinite solution is

$$G_{1}(r,\tau )=\frac{3\mu_{s}^{\prime }}{4\pi }\{\frac{\exp(-k_{D}r_{1})}{r_{1}}-\frac{\exp(-k_{D}r_{2})}{r_{2}}\},$$ (4)

where $k_{D}^{2}=3\mu_{s}^{\prime }\mu_{a}+6\mu_{s}^{\prime 2}k_{0}^{2}\mathrm{BFI}\tau$, $r_{1}=\sqrt{r^{2}+z_{0}^{2}}$, $r_{2}=\sqrt{r^{2}+{\left(z_{0}+2z_{b}\right)}^{2}}$, $k_{0}=2\pi n/\lambda$, n is the tissue index of refraction (i.e., 1.4), λ is the DCS light wavelength (i.e., 785 nm in the present study), $\mu_{a}$ is the tissue optical absorption coefficient, $\mu_{s}^{\prime }$ is the tissue reduced-scattering coefficient, $z_{0}\approx 1/(\mu_{a}+\mu_{s}^{\prime })$ is the effective depth of the source, $z_{b}=2D(1+R_{\mathrm{eff}})/(1-R_{\mathrm{eff}})$ is the distance at which the fluence rate extrapolates to zero, $D=1/[3(\mu_{a}+\mu_{s}^{\prime })]$ is the diffusion constant, and $R_{\mathrm{eff}}$ is an effective Fresnel reflection coefficient. For the present study, the index of refraction of the optical probe was approximately equivalent to the tissue, i.e., $R_{\mathrm{eff}}=0$, and we concurrently measured $\mu_{a}$ and $\mu_{s}^{\prime }$ with TR-NIRS (see Sec. 7 and Appendix 2).

**Fig. 2:**
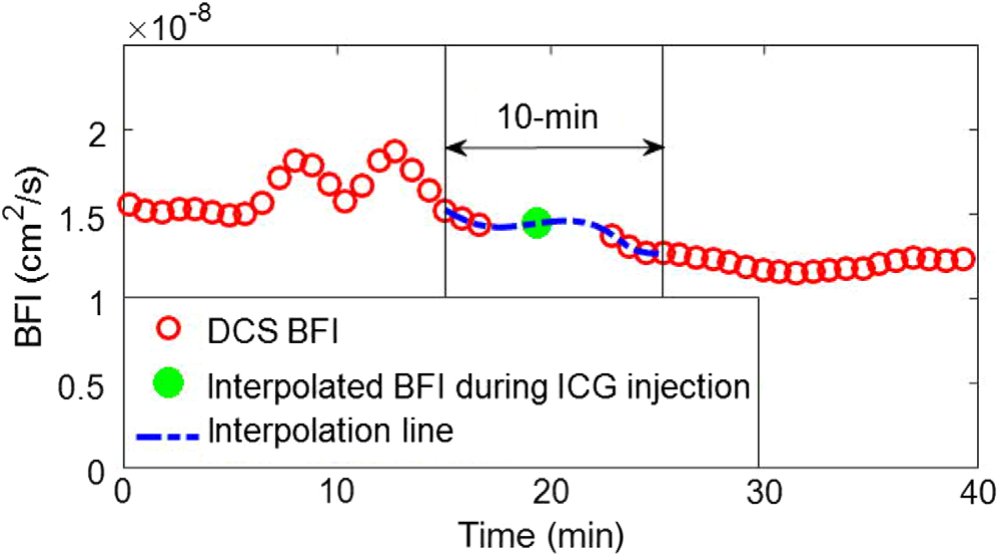
An example showing the use of spline interpolation to estimate ${\mathrm{BFI}}_{0}$, i.e., the BFI at the time of ICG bolus injection. The red circles are continuously measured BFI*s* prior to and after the ICG injection. The dashed blue line is calculated by spline interpolation of the BFI*s* measured with DCS across the 10-min interval starting 5 min before the injection. The filled green circle is the interpolated BFI at the ICG bolus injection time.

Although the DCS BFI has nontraditional units (${\mathrm{cm}}^{2}/s$) for flow, BFI has been shown to be directly proportional to tissue BF, i.e., BF=γ·BFI, where γ is a constant; this relationship has been observed by comparison with numerous independent methods.^23^^,^^53^ γ depends on the nature (e.g., arterial/capillary/venule blood volume fractions) and geometry of the microvasculature underneath the probe, tissue heterogeneities underneath the probe, and tissue optical properties. The DCS calibration coefficient γ for the brain (in our geometry) enables conversion of BFI to absolute CBF units, i.e.,

$$\gamma ={\mathrm{CBF}}_{0}/{\mathrm{BFI}}_{0},$$ (5)

where ${\mathrm{CBF}}_{0}$ and ${\mathrm{BFI}}_{0}$ are the CBF measured by DCE-NIRS and the BFI measured by DCS at the time of ICG bolus administration, respectively. The calibration coefficient γ has units of $\left[(\mathrm{mL}\cdot 100\;g^{-1}\cdot \min^{-1})/({\mathrm{cm}}^{2}/s)\right]$. In practice, the DCS measurement was not acquired simultaneously with the DCE-NIRS measurement. Therefore, ${\mathrm{BFI}}_{0}$ at the time of ICG bolus administration was estimated from spline interpolation (implemented in MATLAB R2016a, Mathworks, Natick, Massachusetts) of the DCS BFI measurements made within the 10-minute interval encompassing the ICG bolus injection (Fig. 2). Alternatively, ${\mathrm{BFI}}_{0}$ can also be interpolated (with similar accuracy) by averaging two BFI measurements taken before and after ICG bolus injection. Absolute DCS BF is achieved by multiplying the measured BFI*s* and γ at all other times during optical monitoring.

The primary goal of this clinical study is to determine and characterize γ in critically brain-injured adult patients using independent measurements of CBF with DCE-NIRS at multiple points in time. A secondary goal is to investigate the accuracy of direct absolute CBF calibration by applying this γ coefficient to DCS-measured BFI from another group of brain-injured patients.

### 2.3 Instrumentation

Our custom-built instrument consists of a TR-NIRS module for measurement of tissue optical properties ($\mu_{a}$ and $\mu_{s}^{\prime }$) and ICG tissue concentration, and a DCS module for measurement of CBF (i.e., BFI). The TR-NIRS light source is a commercial supercontinuum fiber laser (SuperK Extreme EXR-20, NKT Photonics Inc., Morganville, New Jersey) that emits short white-light pulses (400 to 2400 nm, seed pulse width ∼5  ps) at a repetition rate of 78 MHz. The fiber laser was connected to an acousto-optic tunable filter (SuperK Cross, NKT Photonics Inc.) for programmable selection of specific wavelengths. The spectral width for 808 nm is 11 nm, and the switching time between the wavelengths is 200 ms. This light was delivered to tissue via a SuperK Connect fiber delivery system (FD7, NKT Photonics Inc.). Two hybrid single-photon sensitive photomultiplier tubes (PMA hybrid 50, Picoquant Photonics Inc., West Springfield, Massachusetts) were connected to a two-channel time-correlated single-photon counting module (HydraHarp 400, Picoquant Photonics Inc.) and were used for TR-NIRS photon time-of-flight measurements (1 ps resolution). The PMA hybrid detectors were equipped with electrically controlled shutters that were open only during TR-NIRS acquisition, and 665-nm long-pass colored glass filters (RG-665, Edmund Optics, Barrington, New Jersey) were installed to block visible room light. Ideally, a bandpass filter should be used to filter fluorescence from the excited ICG circulating in the brain tissue.^44^^,^^54^ However, due to the required switching between instrument operation modes, the fluorescence filter was not applied in this study. To ameliorate fluorescence effects, we calculated absorption changes from the integral of TPSF in Eq. (2) and we utilized carefully selected time-of-flight intervals^44^ wherein fluorescence contributions would be comparatively small.

The DCS light source is a continuous wave, long coherence length (≥8  m) 785-nm diode laser (IBEAM-SMART-785-S-WS with Smartdock fiber coupler, Toptica Photonics Inc., Victor, New York) connected to a fiber-coupled electrically controlled shutter (OZ Optics, Ottawa, Ontario, Canada) for gated light delivery to tissue. DCS measurements of NIR light intensity correlations (10 Hz sampling rate) were made with four arrays of four high-sensitivity single-photon counting avalanche photodiodes (Excelitas SPCM-AQ4C, Pacer LLC, Palm Beach Gardens, Florida) connected to a multiple-τ 16-channel hardware correlator (Correlator.com, Bridgewater, New Jersey) operating in a burst mode.^55^ For more details about DCS and TR-NIRS instrumentation, we refer readers to recent reviews.^22^^,^^51^^,^^56^^,^^57^

#### 2.3.1 Optical probe

**Fig. 3:**
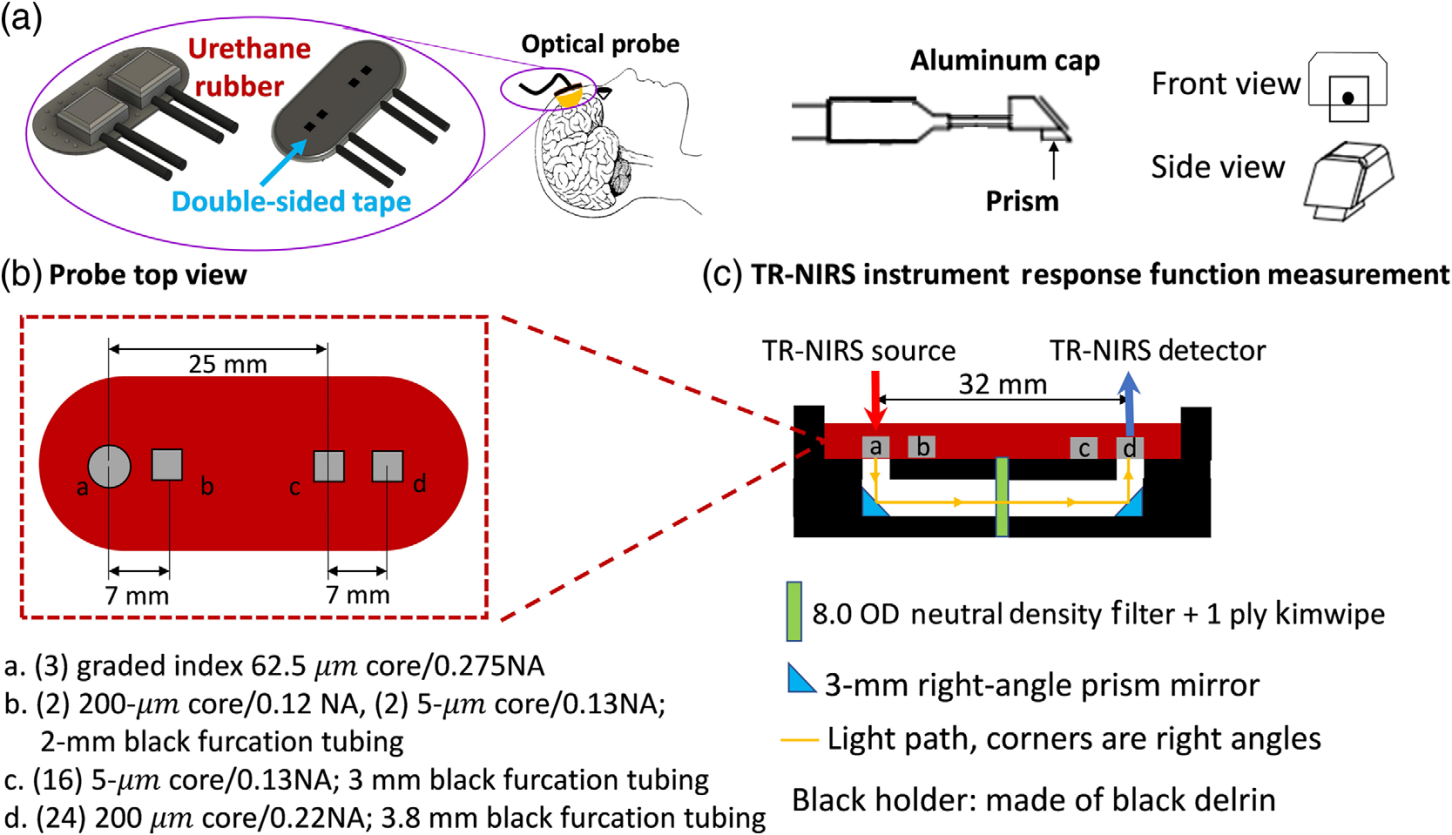
Schematic of the optical probe used for TR-NIRS measurement of tissue optical properties and DCS measurement of BFI (see main text for details). (a) The optical probe consists of prism-coupled fiber bundles embedded in urethane rubber (the prism-fiber connection is protected by an aluminum cap depicted on the right). It is secured to the forehead superior to the frontal sinuses with double-sided tape on the bottom and medical tape around the top (see Sec. 2.3.1). (b) The optical probe uses four fiberoptic bundles (labeled a, b, c, d) for TR-NIRS measurements at 0.7- and 3.2-cm SDS, and DCS measurements at 0.7- and 2.5-cm SDS. In this paper, a semi-infinite tissue model of the head was utilized for the long TR-NIRS and DCS SDS measurements (see Secs. 2.1.1, 2.2, and 4). (c) Schematic of the TR-NIRS IRF measurement (side view; see Sec. 2.3.2). As the urethane rubber mold could not be bent 90 deg to place the source (position a) and detector (position d) prisms in direct contact, right-angle prism mirrors were used instead to direct the light from source to detector.

The optical probe used four 4-m long optical fiber bundles terminated with 3-mm right-angle prisms (MRA03-E03, Thorlabs, Newton, New Jersey) for tissue measurement (Fig. 3). The fiberoptic bundles were assembled by Fiberoptic Systems, Inc. (Simi Valley, California). Light emitted from the TR-NIRS and DCS lasers was delivered to the same location on the tissue via the source bundle [labeled a in Fig. 3(b)], i.e., a bundle of three graded index multimode fibers (62.5-μm core/0.275 NA; GIF625, Thorlabs); 1 for TR-NIRS, 1 for DCS, and 1 extra.

For the DCS long source–detector separation (SDS) measurement, light emerging from the tissue at a distance r=2.5  cm from the source was detected with a bundle of 16 single-mode fibers [5-μm core/0.13 NA, 780 HP, Thorlabs, labeled c in Fig. 3(b)]; 15 of these fibers were connected to detection channels in the DCS instrument (1 fiber per channel). The 15 independent intensity autocorrelation functions acquired in parallel (at the “same” detector position) were subsequently averaged together to improve signal-to-noise ratio.

For the TR-NIRS long SDS measurement, light emerging from the tissue at a distance r=3.2  cm from the source was detected with a bundle of 24 multimode fibers [200-μm core/0.22 NA, Fiberoptic Systems, labeled d in Fig. 3(b)] connected to a PMA hybrid detector. The optical probe also contained a hybrid TR-NIRS/DCS detection bundle [labeled b in Fig. 3(b)] for TR-NIRS and DCS measurement at 0.7-cm SDS. Following a procedure described elsewhere, the optical fiber bundles were embedded at precise positions in urethane rubber.^28^ Note, a first-generation optical probe (rather than the standard probe) was employed for some of the measurements in one patient; in this patient, the source–detector distance may have varied slightly across monitoring days and would have contributed to larger-than-normal variations in calibration.

The optical probe was secured to the forehead superior to the frontal sinuses with sweat-resistant double-sided tape (in contact with skin: #1522, 3M Health Care, St. Paul, Minnesota; in contact with probe: #9917, 3M) on the bottom of the probe (four holes were cut in the tape at the four prism locations, such that the tape did not cover the prisms), and medical tape (Medipore Dress-It, #2954, 3M) around the edges of the top of the probe. Further, a black cloth that spanned ∼2 to 3 cm from the edges of the probe was taped down to block stray light.

In this paper, a semi-infinite tissue model of the head was utilized for the long TR-NIRS and DCS SDS measurements. The limitations of the semi-infinite model are explored in Sec. 4.

#### 2.3.2 Instrument response function measurement

The urethane rubber securing the prisms in place could not be bent to place the source (position a) and detector (position d) prisms in direct contact (Fig. 3). Therefore, the TR-NIRS IRF was measured using right-angle prism mirrors to direct the source light to the detector as shown in Fig. 3(c). To attenuate light properly and provide a wide angular distribution of the light,^58^ an 8.0 optical density (OD) neutral density filter (two sheets, 54-459, Edmund Optics) and one one-ply sheet of kimwipe (KW32, Thorlabs), i.e, as a diffuser, were placed in the middle of the light path. (Note, for fibers with larger NA, it is recommended that the diffuser be attached close to the tip of the detection fibers.) The FWHM bandwidth of the IRF was 190 ps at 14 mW output power emerging from the source fiber (808-nm wavelength). The FWHM of the IRF is narrow compared with the typical width of the TPSF from brain tissue (i.e., FWHM≈830  ps at 3.2-cm SDS). The IRF measurement was used to define the launch time of the incident source pulse in the calculation of the absorption change induced by ICG injection; it was also used to extract optical properties at 786 nm from the TR-NIRS measurement which, in turn, was used in the DCS fittings to derive DCS BFI.

### 2.4 Monitoring Protocol

**Fig. 4:**
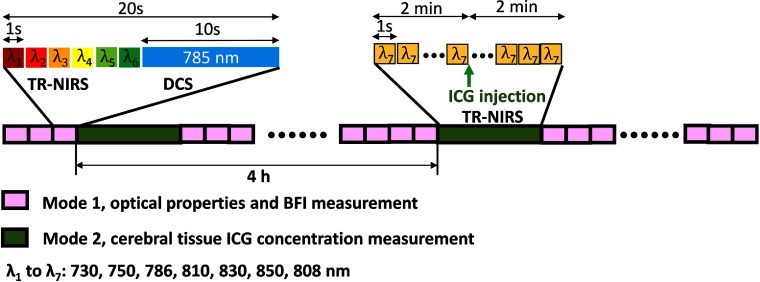
Schematic of monitoring protocol (Sec. 2.4). During each monitoring day, TR-NIRS and DCS continuously measured tissue optical properties ($\mu_{a}$ and $\mu_{s}^{\prime }$) and BFI (pink boxes, instrument mode 1). For the absolute CBF measurement via ICG bolus injection, continuous single-wavelength (808 nm) TR-NIRS measurements were made to track the kinetics of the ICG bolus through the TR-NIRS sampled cerebral tissue (green boxes, instrument mode 2).

Optical data acquisition was controlled with a custom-written software in the Labview environment (National Instruments, Austin, Texas). The instrument was used in two modes of operations (Fig. 4): (1) TR-NIRS measurement of absolute tissue optical absorption ($\mu_{a}$) and tissue optical reduced scattering ($\mu_{s}^{\prime }$) coefficients at six wavelengths (730, 750, 786, 810, 830, 850 nm; 800-ms exposure time per wavelength) with DCS measurement (10 s exposure time) of BFI and (2) ICG concentration measurement. In the first mode, the instrument sequentially interleaved TR-NIRS tissue measurements at six wavelengths with a DCS measurement (10 s exposure). This enabled us to derive $\mu_{a}$ and $\mu_{s}^{\prime }$, and BFI every 20 s. The multispectral measurements of tissue optical absorption, which were made for the estimation of total hemoglobin concentration and tissue oxygen saturation, are not the focus of this paper. Only the optical properties from the 786-nm wavelength were used in this study to extract DCS BFI.

In the second mode, cerebral tissue ICG concentration and arterial ICG concentration were monitored with single-wavelength TR-NIRS (808 nm, 1 Hz) and customized dye densitometry (see Sec. 6 and Appendix 1), respectively, to track the kinetics of ICG passage through the brain following intravenous bolus injection.^19^^,^^20^ A 2-min baseline measurement was made prior to ICG bolus injection, and 2-min continuous monitoring was carried out after the injection. After completion of this procedure, the instrument was switched back to the first operation mode. Two ICG bolus injections, spaced 4 h apart, were given per subject monitoring day.

### 2.5 Patient Population and Study Design

Seven adult patients in the neurointensive care unit were studied (six males, one female, age 43.9±13.5 years). The enrolled patients were diagnosed with traumatic brain injury (N=3), intracerebral hemorrhage (N=2), or postischemic encephalopathy (N=2). Patients were recruited through the Departments of Neurology and Anesthesiology and Critical Care with the Neurosurgery Clinical Research Division at the Hospital of the University of Pennsylvania. Written consent for all subjects was provided by legally authorized representatives, and all protocols/procedures were approved by the Institutional Review Board at the University of Pennsylvania, which adheres to the guidelines of the Common Rule and the Food and Drug Administration’s Institutional Review Board’s human subject regulations. Approximately four days of subject monitoring for eight hours on each day were targeted, though this goal was not always feasible due to clinical care needs.

**Fig. 5:**
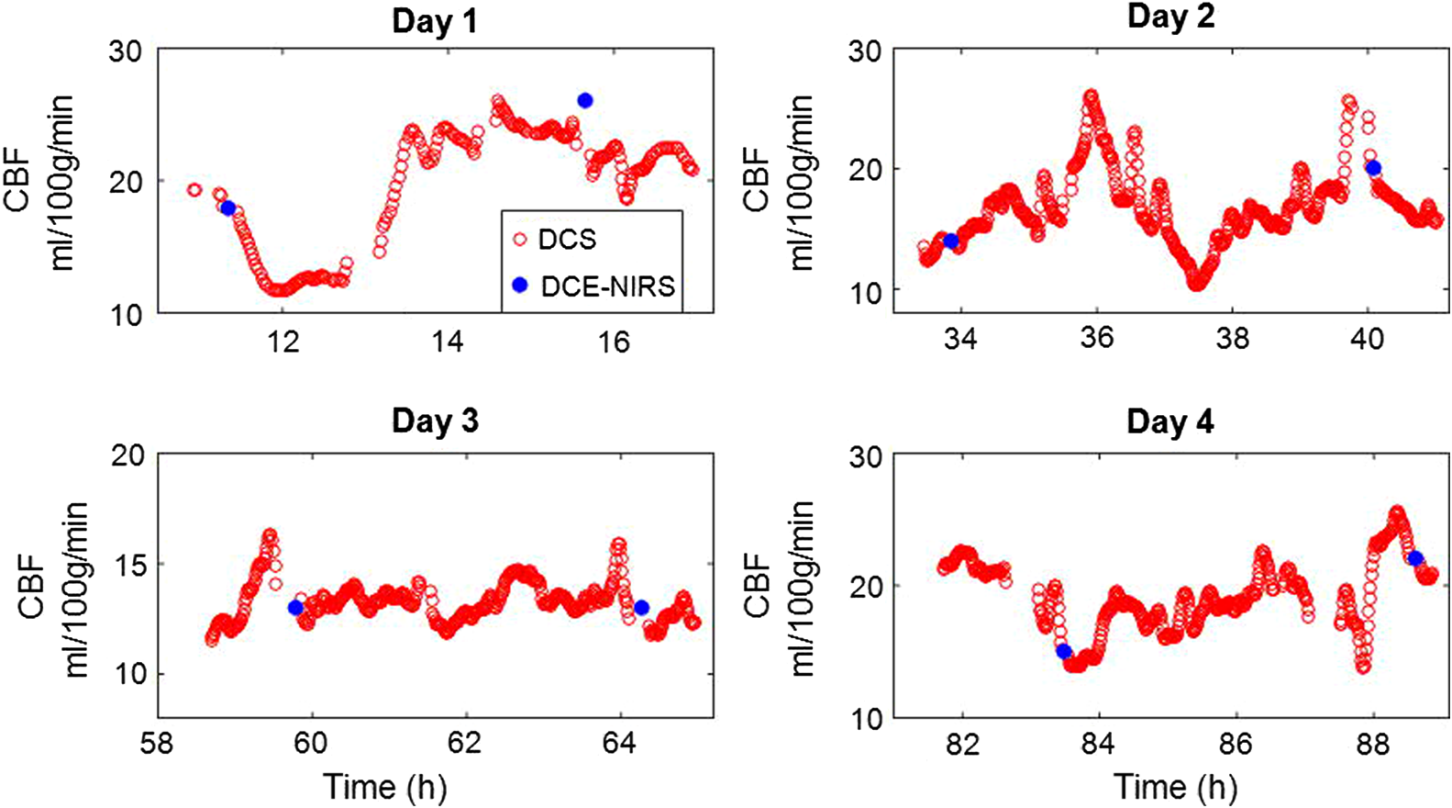
Continuous calibrated absolute CBF measurements with DCS (red dots) and DCE-NIRS measurement of absolute CBF via intravenous ICG bolus injection (blue dots) in an exemplar patient diagnosed with traumatic brain injury. Here, the DCS measurements were calibrated with the first DCE-NIRS CBF measurement. This patient was continuously monitored for 4 days. For each monitoring day, two ICG bolus injections were administered 4 h apart.

On each subject monitoring day, the optical probe was secured above the fronto-parietal cortex (see Sec. 2.3.1) and the customized dye densitometer was secured to the subject’s index finger (see Sec. 6 Appendix 1). Approximately 8 h of continuous optical measurements were acquired (as described in Sec. 2.4), and two intravenous ICG bolus injections (0.1 or 0.2  mg/kg; <2  s per injection) were administered ∼4  h apart to calibrate the DCS BFI for absolute CBF measurement (see Fig. 5 for an example).^19^^,^^20^ Note, ICG is cleared from the body in ∼20  min, and thus the calibration data could in principle have been taken more often. The 4-h time window we chose represents a compromise between the technical challenges of ICG injection, i.e., patient consent, ICG costs, and clinical personnel/workflow, against the need to generate adequate statistics to ascertain calibration. Thus, two calibration coefficients, $\gamma_{1}={\mathrm{CBF}}_{0,1}/{\mathrm{BFI}}_{0,1}$ and $\gamma_{2}={\mathrm{CBF}}_{0,2}/{\mathrm{BFI}}_{0,2}$, were obtained for a single patient across a single day. Here, the subscripts 1 and 2 denote first and second time points, respectively, of the two ICG injections. These calibration coefficients were compared to ascertain the stability of the ICG calibration technique according to Eq. (5). Note, due to clinical-care needs, for some of the monitoring days only 1 ICG injection was feasible.

We made 44 ICG injections across 24 monitoring days in the seven critically brain-injured patients. Of these 44 injections, absolute CBF could not be measured for eight of them; seven of these eight were due to the insufficient data quality in the $C_{a}(t)$ measurement, and one of the eight was due to significant heart rate increase during ICG injection (i.e., heart rate increased from 95 to 133 bpm) such that CBF was not constant during ICG bolus. Of the 24 monitoring days, continuous optical monitoring with two ICG boluses administered 4 h apart was feasible in 13 cases. Note, five of these monitoring days were for the cases wherein absolute CBF for one or two of the injections could not be assessed, for the reasons described above. For four of these monitoring days, it was only possible to give patients one ICG bolus. In the remaining 2 days, the optical probe needed to be removed for a clinical procedure.

## 3 Results

### 3.1 DCS Single-Day Calibrations

**Fig. 6:**
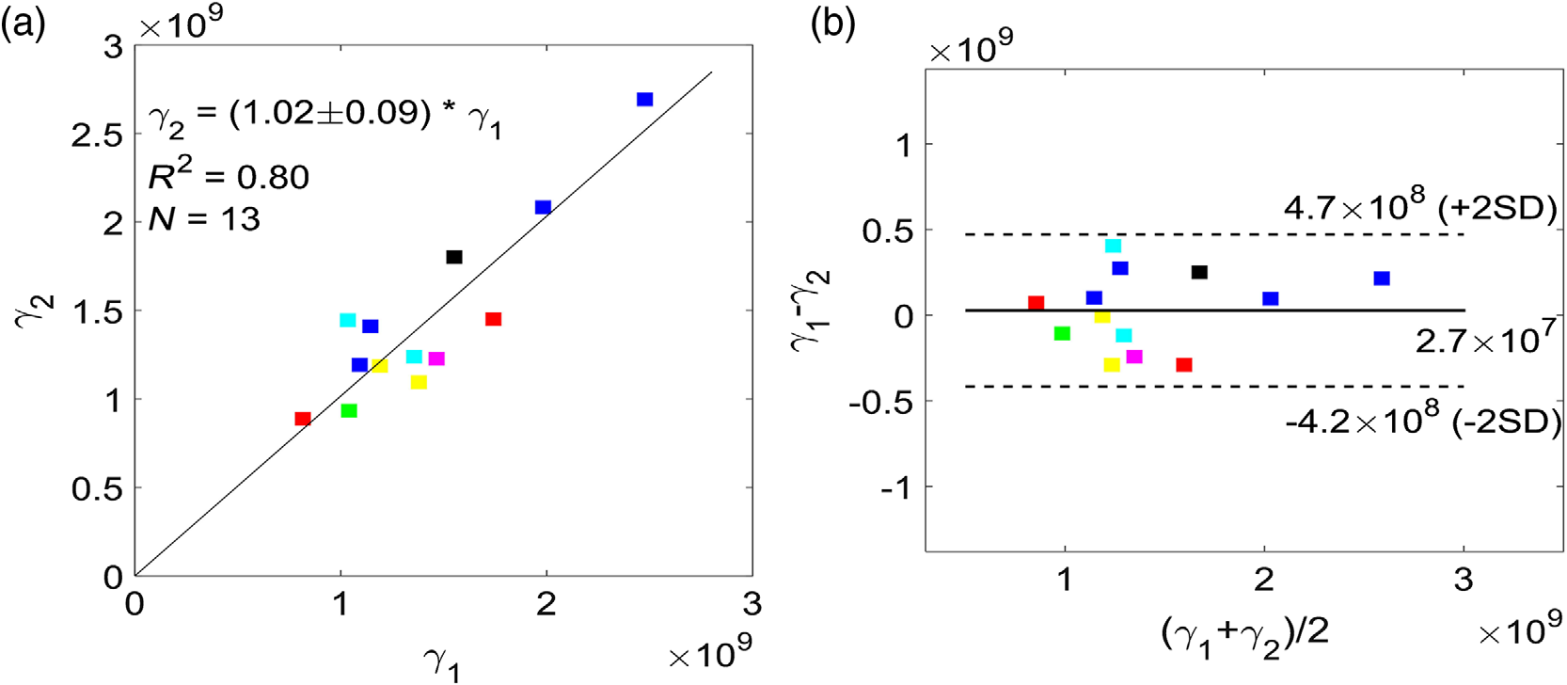
(a) DCS calibration coefficients [unit: ($\mathrm{mL}\cdot 100\;g^{-1}\cdot \min^{-1})/({\mathrm{cm}}^{2}/s)$]from first ICG bolus injection (horizontal axis) and second injection (vertical axis) across N=13 monitoring days in 7 patients. Bolus injections are 4 h apart. The black line represents the best linear fit to the data with the intercept forced to be zero ($R^{2}=0.80$, slope±95CI=1.02±0.09). (b) Bland–Altman plot of the difference in calibration coefficients from two ICG injections versus the mean of the two coefficients. The solid horizontal line indicates the mean difference between the two parameters computed across the study population, which is not significantly different from zero (p=0.67 with Wilcoxon rank sum test). The dotted line indicates 95% CI limits for agreement. Different colors in (a) and (b) represent different patients.

We calibrated DCS BF twice a day using the DCE-NIRS technique during the patient monitoring days wherein continuous BFI monitoring between doses was feasible (N=13). Figure 6 shows comparison of two calibration coefficients, $\gamma_{1}$ and $\gamma_{2}$, for each patient across a single monitoring day. We observed a largely linear correlation ($R^{2}=0.80$, N=23, P<0.01) and a slope close to unity (slope=1.02±0.09, here the error is the 95% confidence interval [CI]) [Fig. 6(a)]. Additionally, Bland–Altman analysis of these data [Fig. 6(b)] reveals a mean difference in the two calibration coefficients of $2.7\times 10^{7}\;(\mathrm{mL}\cdot 100\;g^{-1}\cdot \min^{-1})/({\mathrm{cm}}^{2}/s)$, which is not significantly different from zero (p=0.67 with Wilcoxon rank sum test). The limits of agreement between the two calibration coefficients [dashed lines in Fig. 6(b)], within which 95% of the differences reside are $4.7\times 10^{8}$ and $-4.2\times 10^{8}\;(\mathrm{mL}\cdot 100\;g^{-1}\cdot \min^{-1})/({\mathrm{cm}}^{2}/s)$. This 95% CI ($\pm 4.4\times 10^{8}$) divided by the mean of the two calibration coefficients $\left[1.42\times 10^{9}\;(\mathrm{mL}\cdot 100\;g^{-1}\cdot \min^{-1})/({\mathrm{cm}}^{2}/s)\right]$ is ±31%. Thus, the DCE-NIRS technique for DCS BF calibration is quite stable across a single monitoring day for a single patient.

We used the coefficient of variation (CV) to evaluate the stability of same-day calibration within a single subject. The CV across a single monitoring day was defined as $\left|\gamma_{2}-\gamma_{1}|/0.5(\gamma_{2}+\gamma_{1}\right)$. Across 13 monitoring days, CV was 6.7±4.1% (mean±SD). This result indicates that if each patient receives one-time DCS calibration for absolute CBF, then absolute CBF is expected to exhibit ∼7% variation after ∼4  h.

### 3.2 DCS Calibration across Multiple Days

**Fig. 7:**
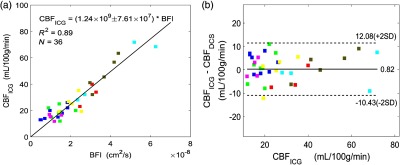
(a) Absolute BF measured by DCE-NIRS technique (vertical axis) compared with the BFI measured with DCS (horizontal axis) across N=36 measurements. The black line represents the best linear fit to the data with the intercept forced to be zero ($\text{slope}=(1.24\pm 0.08)\times 10^{9}$, $R^{2}=0.89$, p<0.01 with F-test). (b) Bland–Altman plot of the difference in absolute CBF measurements from DCS and DCE-NIRS techniques. The solid horizontal line indicates the mean difference between the two parameters computed across the study population, which is not significantly different from zero (p=0.47 with Wilcoxon rank sum test). The dotted line indicates 95% CI limits for agreement. Different colors in (a) and (b) represent different patients.

The absolute CBF measured with DCE-NIRS across all patients and all monitoring days was significantly correlated with the absolute BFI measured with DCS [$R^{2}=0.89$, N=36, P<0.01 with F-test, see Fig. 7(a)]. Note, as DCE-NIRS and DCS sample similar brain volumes, and since both data analyses employed a semi-infinite head model, we forced the intercept to be zero in the linear regression analysis. We can derive a “best” DCS calibration coefficient from the slope (±95% CI) of the best-fit line, i.e., $\gamma =(1.24\pm 0.08)\times 10^{9}\;(\mathrm{mL}/100\;g/\min)/({\mathrm{cm}}^{2}/s)$. We then applied this estimate to all measurements in the same data to convert the DCS BFI to absolute CBF, i.e., ${\mathrm{CBF}}_{\mathrm{DCS}}=\gamma \times \mathrm{BFI}$, and we performed a Bland–Altman analysis to assess error in ${\mathrm{CBF}}_{\mathrm{DCS}}$. The Bland–Altman analysis reveals that a mean difference in CBF measured by two techniques, i.e, by DCS and DCE-NIRS, of 0.82  mL/100  g/min, is not significantly different from zero (p=0.47 with Wilcoxon rank sum test) [Fig. 7(b)]. The limits of agreement between the two CBF measurement techniques [dashed lines in Fig. 7(b)], within which 95% of the differences resides, are −10.43 and $12.08\;(\mathrm{mL}\cdot 100\;g^{-1}\cdot \min^{-1})$. The 95% CI [i.e., $\pm 11\;(\mathrm{mL}\cdot 100\;g^{-1}\cdot \min^{-1})$] divided by the mean ${\mathrm{CBF}}_{\mathrm{ICG}}$ [i.e., $29\;(\mathrm{mL}\cdot 100\;g^{-1}\cdot \min^{-1})$] is ±38%. Thus, a single ${\mathrm{CBF}}_{\mathrm{DCS}}$ measurement is expected to be within 38% of the actual CBF.

We used CV to evaluate the stability of calibration across multiple monitoring days within a single subject. Here, a single calibration coefficient was obtained for each monitoring day (i.e., the mean of $\gamma_{1}$ and $\gamma_{2}$, or just $\gamma_{1}$ if only one ICG injection was made). The CV coefficient for each patient is defined as the standard deviation of this calibration coefficient for each patient across multiple monitoring days divided by their mean. Across seven patients, CV was 21±14% (mean±SD). This finding indicates that if each patient receives a one-time DCS calibration for absolute CBF monitoring, then absolute CBF is expected to exhibit ∼20% variation in the following days.

### 3.3 Testing Calibrated-DCS for CBF of Brain-Injured Patients in Prior Study

The strong linear relationship between CBF measured with DCE-NIRS and DCS BFI across multiple monitoring days (Fig. 7) suggests the potential for using DCS BFI directly as a measure of absolute CBF, i.e., ${\mathrm{CBF}}_{\mathrm{DCS}}=(1.24\times 10^{9})\times \mathrm{BFI}$ (see Fig. 7). We tested this approach in an independent dataset. Specifically, we compared ${\mathrm{CBF}}_{\mathrm{DCS}}$ obtained from previous DCS BFI measurements in seven brain-injured patients (i.e., carried out by our groups and published in Ref. 21) to concurrent measurements of absolute CBF made with the XeCT technique, i.e., ${\mathrm{CBF}}_{\mathrm{XeCT}}$. The previous study^21^ made BFI measurements on both sides (bilateral) of the forehead using a DCS SDS of 2.5 cm and employing a semi-infinite homogeneous tissue model for the head (i.e., the same theoretical model as used in the present paper). The ${\mathrm{CBF}}_{\mathrm{XeCT}}$ was obtained from averaging of the voxels on the cerebral cortex under the optical probes.^21^ During this prior study, pressors were administered to increase blood pressure, and a second concurrent set of bilateral BFI and ${\mathrm{CBF}}_{\mathrm{XeCT}}$ measurements was obtained for each patient. Thus, 24 concurrent BFI and ${\mathrm{CBF}}_{\mathrm{XeCT}}$ measurements were obtained across seven patients.

**Fig. 8:**
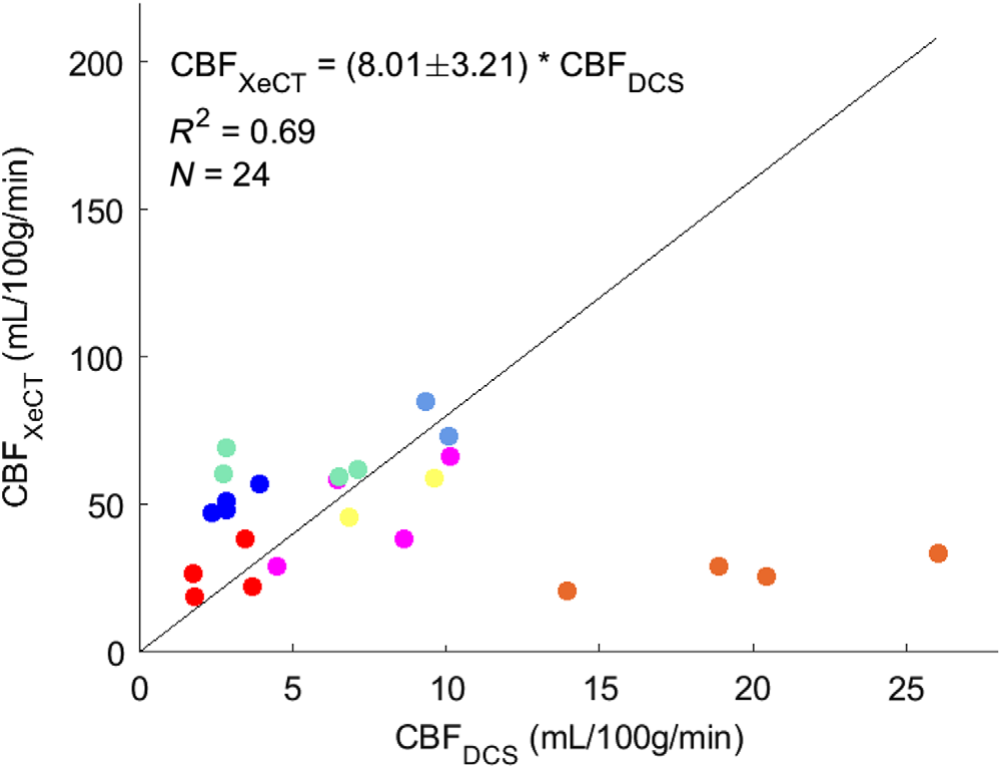
Absolute CBF measured by XeCT technique (vertical axis) compared with the CBF measured with DCS (horizontal axis) across N=24 measurements. The black line represents the best linear fit to the data with the intercept forced to be zero (slope=8.0±3.2, $R^{2}=0.69$, p<0.01 with F-test). Different colors represent different patients.

We converted the prior BFI data to ${\mathrm{CBF}}_{\mathrm{DCS}}$. A linear mixed-effects model was defined wherein ${\mathrm{CBF}}_{\mathrm{XeCT}}$ is the response variable, ${\mathrm{CBF}}_{\mathrm{DCS}}$ is the predictor variable, and patient ID is the grouping variable. The model was analyzed by the MATLAB^®^ function fitlme, and the results indicate that ${\mathrm{CBF}}_{\mathrm{XeCT}}$ was significantly correlated with the ${\mathrm{CBF}}_{\mathrm{DCS}}$ ($R^{2}=0.69$, N=24, P<0.01 with F-test) (Fig. 8). From the slope (±95% CI) of a linear mixed-effect model, we estimate (8.0±3.2)-fold difference between ${\mathrm{CBF}}_{\mathrm{DCS}}$ converted from DCS BFI and ${\mathrm{CBF}}_{\mathrm{XeCT}}$, i.e., ${\mathrm{CBF}}_{\mathrm{XeCT}}=(8.0\pm 3.2)\times {\mathrm{CBF}}_{\mathrm{DCS}}$. These systematic differences between DCS-calibrated CBF and ${\mathrm{CBF}}_{\mathrm{XeCT}}$ can be understood, in part, to be a consequence of the semi-infinite model for brain (see below).

## 4 Discussion

To our knowledge, the present study is the first report of the calibration coefficient for absolute CBF monitoring with DCS in critically brain-injured adult patients. Several important previous studies have demonstrated that the same technique can be used to convert DCS data into absolute CBF on newborn piglets^19^ and babies.^36^ However, none have tested the stability of the calibration technique across long time periods. In this paper, we determine the stability of the calibration technique and investigate the utility of absolute CBF monitoring by direct DCS measurement throughout the course of treatment. Note, the instrumentation represents a state-of-the-art TR-NIRS and DCS system for various reasons, but such a complex TR-NIRS is not necessary for continuous absolute CBF monitoring.

In regard to stability, we found that the DCS calibration coefficient, γ, remains constant over at least 4 h of monitoring; good agreement was observed between the two daily calibration coefficients determined from two ICG bolus injections administered 4 hours apart [Fig. 6(a)]. Although only 4-h intervals were directly tested, γ should remain stable for longer intervals, provided probe contact is stable. Intrasubject variation in γ across multiple days, with probe removal and reapplication between daily monitoring sessions, was larger than the same-day results, with a mean coefficient of variation across subjects of 21%±14% (Sec. 3.2). Thus, our results indicate that when removing the probe and reapplying the probe to roughly the same location the next day, a change in γ of ∼20% can be expected (at least for the present protocols). Intrapatient variability, however, is sometimes observed to be larger across monitoring days. One large variation (marked with blue) was caused by use of first- and second generations of the probe (which varied slightly), and resultant variations in source–detector distance across monitoring days can introduce discrepancies into the calibration coefficients. In addition, some patients were observed to experience excessive sweating during the monitoring, requiring extra tape, and wrapping to secure the probe; this effect, in turn, could increase probe pressure and cause the calibration coefficient to vary. Generally, factors such as variation in the physiological state of underlying brain tissue, probe location, and probe pressure against the head can induce variation in the calibration coefficient. For this reason, our study utilized daily calibration, and the study suggests that daily calibration of DCS is optimal.

### 4.1 Estimation of Absolute CBF from DCS BFI Measurements

In regard to using the DCS BFI as a direct measure of absolute CBF, we observed a strong linear correlation ($R^{2}=0.89$) between absolute CBF measured with DCE-NIRS and DCS BFI [Fig. 7(a)]. Using the slope of the best fit line as the γ coefficient, estimation of absolute CBF from the DCS BFI is ${\mathrm{CBF}}_{\mathrm{DCS}}=(1.24\times 10^{9})\mathrm{BFI}$. Via Bland–Altman analysis, the mean difference between ${\mathrm{CBF}}_{\mathrm{DCS}}$ and CBF measured with DCE-NIRS, i.e., ${\mathrm{CBF}}_{\mathrm{ICG}}$, was not significantly different from zero [p=0.47 with Wilcoxon rank sum test, Fig. 7(b)]. The variability between ${\mathrm{CBF}}_{\mathrm{DCS}}$ and ${\mathrm{CBF}}_{\mathrm{ICG}}$, i.e., defined as the 95% CI of their difference divided by the mean ${\mathrm{CBF}}_{\mathrm{ICG}}$ across measurements, was 38%. Thus, conservatively, our results suggest that ${\mathrm{CBF}}_{\mathrm{DCS}}$ should be within 40% of the CBF, as measured by DCE-NIRS. Note that the DCS calibration coefficient of $\gamma =1.24\times 10^{9}\;[(\mathrm{mL}\cdot 100\;g^{-1}\cdot \min^{-1})/({\mathrm{cm}}^{2}\cdot s^{-1})$] obtained for this cohort is comparable with the DCS calibration coefficient previously obtained in piglets with the same DCE-NIRS technique,  i.e., $1.14\times 10^{9}\;[(\mathrm{mL}\cdot 100\;g^{-1}\cdot \min^{-1})/({\mathrm{cm}}^{2}\cdot s^{-1})]$, $R^{2}=0.89$.^19^

### 4.2 CBF Estimates Obtained with Semi-Infinite Head Model Underestimates True CBF

We used the DCS calibration coefficient, $\gamma =1.24\times 10^{9}\;[\mathrm{mL}\cdot 100\;g^{-1}\cdot \min^{-1})/({\mathrm{cm}}^{2}/s)])$, extracted from this cohort to convert prior BFI data in brain injured patients (N=7) to absolute CBF (Sec. 3.3), which again, is denoted as ${\mathrm{CBF}}_{\mathrm{DCS}}$. In comparison with concurrent CBF measurements obtained with XeCT, i.e., ${\mathrm{CBF}}_{\mathrm{XeCT}}$, linear regression analysis showed that although ${\mathrm{CBF}}_{\mathrm{DCS}}$ and ${\mathrm{CBF}}_{\mathrm{XeCT}}$ were correlated ($R^{2}=0.69$), ${\mathrm{CBF}}_{\mathrm{DCS}}$ underestimated ${\mathrm{CBF}}_{\mathrm{XeCT}}$ by roughly a factor of 8 (i.e., slope±95%CI=8.0±3.2). We can understand this systematic deviation (see below), but we also note that the early work had many limitations that could have weakened correlations. For example, the earlier paper assumed rather than measured tissue optical properties (i.e., $\mu_{a}=0.11\;{\mathrm{cm}}^{-1}$ and $\mu_{s}^{\prime }=12\;{\mathrm{cm}}^{-1}$) to compute BFI; this approach translates to errors in BFI.^19^^,^^59^^,^^60^ The overall underestimation, however, largely arises from using the semi-infinite homogeneous head model for determining γ and BFI (Secs. 2.1 and 2.2).

It is known that the semi-infinite model underestimates localized CBF changes. In previous measurements of humans performing a finger tapping task, CBF relative changes computed with the semi-infinite model underestimated the true changes by a factor of 5.^61^ This result was subsequently confirmed in realistic simulations of the head.^62^ For absolute CBF estimates on healthy adult subjects with DCE-NIRS, mean CBF with the semi-infinite model was 8.3  mL/100  g/min, which was believed to have large underestimation compared with values obtained by PET, i.e., a factor of 6.^38^^,^^63^^,^^64^ In two-layer simulations of the head, DCE-NIRS CBF measurements obtained from application of the semi-infinite model to continuous-wave NIRS data also underestimated the true CBF by around eightfold.^63^

To further elucidate this issue, we performed two-layer simulations of the head to assess underestimation of DCE-NIRS CBF measurements obtained from application of the semi-infinite model to simulated two-layer time-resolved NIRS data (details in Sec. 8 and Appendix 3). Overall, we found that the underestimation of CBF with the semi-infinite model ranged from 3.5 to 6.5, with top layer thickness being the most sensitive parameter for different levels of underestimation (Sec. 8 and Appendix 3). Thus, the underestimation of ${\mathrm{CBF}}_{\mathrm{DCS}}$ compared with CBF measured from XeCT (slope=8.0±3.2, N=24, $R^{2}=0.69$, Sec. 3.3) partially overlapped with the range of underestimation found from this computer simulation.

### 4.3 Limitations

The use of the semi-infinite model for estimation of absolute CBF with DCE-NIRS is the most significant limitation for obtaining calibration coefficients in the present paper. Note, this limitation does not alter the paper’s findings and conclusions about measurement stability. Different algorithms beyond the semi-infinite model have been proposed to improve the depth sensitivity of TR-NIRS measurements.^20^^,^^65^^–^^69^ One promising approach is the use of changes in the variance moment of the TPSF TR-NIRS measurement for retrieval of ICG concentration.^68^^,^^70^^,^^71^ The linearity between changes in the variance moment of TPSF and changes in absorption holds, however, only when the changes in absorption are sufficiently small.^70^^,^^72^ In simulations (see Sec. 4.2), we find that when the absorption changes are very large due to ICG, then a substantial underestimation of the cerebral absorption change is introduced when extracted by the variance moment analysis (e.g., by ∼72%). To ameliorate this situation in the future, we will include higher order terms related to the absorption change; this method permits retrieval of more accurate absolute CBF and can be applied for bolus injection of ICG at high concentration.^34^ In a different vein, the short source–detector separation data were not used for several reasons. First, the TR-NIR measurement was not able to generate accurate optical properties, because the temporal width of IRF and tissue TPSF curves were too similar (FWHMs are 190 versus 300 ps). Also, we did not consistently obtain information about scalp and skull thickness (under the probe), because the necessary CT scans were not always taken during our optical monitoring session. Given these sources of error, we felt that only the large source–detector separations and semi-infinite head models were justified. In the future, we anticipate that short SDSs for both TR-NIRS and DCS measurement will be used along with a layered DCS model for better computation of cerebral BFI,^28^^,^^73^ and absolute DCS CBF calibration coefficient.

## 5 Conclusion

In this study, we demonstrated the long-term stability of noninvasive absolute CBF monitoring with DCE-NIRS calibrated DCS. The calibration was stable across a single monitoring day, and the variability for each patient across multiple monitoring days was moderate (20% varying between 3% and 40%). The variability in calibration coefficient across multiple monitoring days and multiple subjects was small enough that a strong correlation between CBF measured with DCE-NIRS and DCS BFI was observed. However, for single measurements, deviations of up to 40% from the best fit line of DCE-NIRS CBF to DCS BFI can be an issue for some applications. If feasible, it is best to calibrate DCS with DCE-NIRS prior to every patient monitoring session. Finally, the semi-infinite model estimate of absolute CBF substantially underestimates the true CBF.

## 6 Appendix 1: Principle of Customized ICG Dye Densitometer

For measurement of arterial ICG concentration per volume of blood in the finger, i.e., $C_{\text{finger}}$ (Sec. 2.1), we used a customized dye densitometer that consisted of a two-wavelength (804 and 938 nm) finger sensor (TL-301P, Nihon Kohden, Cambridge, Massachusetts) connected to an integrated analog front-end commercial pulse oximeter circuit board (AFE4400, Texas Instruments, Dallas, Texas), which was in turn connected to a laptop computer. The circuit board cyclically altered illumination of the finger between wavelengths $\lambda_{1}=804\;\mathrm{nm}$ and $\lambda_{2}=938\;\mathrm{nm}$ (5-ms exposure per wavelength), and also recorded their temporal intensities transmitted through the finger, i.e., ${\tilde{I}}_{\left[\lambda_{1}\right]}(t)$ and ${\overset{˜}{I}}_{\left[\lambda_{2}\right]}(t)$, at 100-Hz sampling rate. Here, we introduce the principle of extracting $C_{\text{finger}}(t)$ from these measurements.

First, a finite impulse response digital bandpass filter with a passband frequency from $0.95f_{hr}$ to $1.05f_{hr}$, wherein $f_{hr}$ is the average heart rate across the four-minute interval encompassing the ICG injection (see Fig. 4, mode 2), was constructed in the MATLAB R2016a software environment (designfilt, Mathworks), and applied to ${\tilde{I}}_{\left[\lambda_{1}\right]}(t)$ and ${\overset{˜}{I}}_{\left[\lambda_{2}\right]}(t)$. Hereafter, we will denote the bandpass filtered data as $I_{\left[\lambda_{1}\right]}(t)$ and $I_{\left[\lambda_{2}\right]}(t)$. The modified Beer–Lambert law^74^^,^^75^ was next employed to relate the measured peak-to-peak amplitude of the pulsatile oscillation during the cardiac cycle in the bandpass filtered optical density to the peak-to-peak amplitudes of the pulsatile oscillations in light chromophore concentrations, i.e.,

$${\Delta \mathrm{OD}}_{\left[\lambda \right]}(t)\equiv -\log[\frac{I_{1[\lambda ]}(t)}{I_{2[\lambda ]}(t)}]=[\epsilon_{H_{b}O[\lambda ]}\Delta [H_{b}O](t)+\epsilon_{H_{b}[\lambda ]}\Delta [H_{b}R](t)+\epsilon_{\mathrm{ICG}[\lambda ]}\Delta [\mathrm{ICG}](t)]d_{\left[\lambda \right]},$$ (6)

where t refers to averaged times across single heart beats (e.g., for $f_{hr}=60\;\mathrm{bpm}$, t=0.5  s, 1.5 s, 2.5 s,…); $I_{1[\lambda ]}(t)$ and $I_{2[\lambda ]}(t)$ are the peak and minimum bandpass filtered intensities at wavelength λ, respectively, during the cardiac cycle associated with the heart beat at time t; $\Delta [H_{b}O](t)$, $\Delta [H_{b}R](t)$, and Δ[ICG](t) are the differences between the peak and minimum bandpass filtered oxyhemoglobin, deoxyhemoglobin, and ICG tissue concentrations (per volume of tissue), respectively, during the cardiac cycle at time t; $\epsilon_{H_{b}O[\lambda ]}$, $\epsilon_{H_{b[\lambda ]}}$, and $\epsilon_{\mathrm{ICG}[\lambda ]}$ are the extinction coefficients of oxyhemoglobin, deoxyhemoglobin, and ICG (measured at 6.5  μM concentration in plasma), respectively, at wavelength λ;^45^ and $d_{\left[\lambda \right]}$ is the differential path length at wavelength λ. Assuming that heart rate induced blood volume oscillations are (1) faster than changes in chromophore concentrations (per volume blood) and (2) present only in the arterial compartment, then

$$\Delta [H_{b}O]=S_{a}\Delta [H_{b}T],\;\Delta [H_{b}R]=(1-S_{a})\Delta [H_{b}T],\;\Delta [\mathrm{ICG}]=C_{\text{finger}}\Delta [H_{b}T]/[H_{g}b],$$ (7)

where $S_{a}$ is the arterial oxygen saturation, $\Delta [H_{b}T]$ is the difference between the peak and minimum bandpass filtered total hemoglobin concentration (per volume tissue), and $\left[H_{g}b\right]$ is the total hemoglobin concentration per volume blood. Note, the time-dependence t is implicit for all parameters in Eq. (7). The expression for Δ[ICG] in Eq. (7) is understood from noting that

$$[\mathrm{ICG}]\equiv \frac{\mu_{\mathrm{mol}}\mathrm{ICG}}{V_{\text{tissue}}}=\frac{\mu_{\mathrm{mol}}\mathrm{ICG}}{V_{\text{blood}}}\cdot \frac{V_{\text{blood}}}{V_{\text{tissue}}}=C_{\text{finger}}\cdot \frac{\left[H_{b}T\right]}{\left[H_{g}b\right]}.$$

Substituting Eq. (7) into (6), we obtain

$${\Delta \mathrm{OD}}_{\left[\lambda \right]}=d_{\left[\lambda \right]}[\epsilon_{H_{b}O[\lambda ]}\cdot S_{a}+\epsilon_{H_{b}R[\lambda ]}\cdot (1-S_{a})+\epsilon_{\mathrm{ICG}[\lambda ]}\frac{C_{\text{finger}}}{\left[H_{g}b\right]}]\cdot \Delta [H_{b}T].$$ (8)

Equation (8) is then used to generate a system of two equations that correspond to the two light wavelengths in the sensor ($\lambda_{1}=804\;\mathrm{nm}$ and $\lambda_{2}=938\;\mathrm{nm}$), wherein $\epsilon_{\mathrm{ICG}[\lambda 2]}=0$:

$${\Delta \mathrm{OD}}_{\left[\lambda_{1}\right]}=d_{\left[\lambda_{1}\right]}[\epsilon_{H_{b}O[\lambda_{1}]}\cdot S_{a}+\epsilon_{H_{b}R[\lambda_{1}]}\cdot (1-S_{a})+\epsilon_{\mathrm{ICG}[\lambda_{1}]}\frac{C_{\text{finger}}}{\left[H_{g}b\right]}]\cdot \Delta [H_{b}T],$$ (9)

$${\Delta \mathrm{OD}}_{\left[\lambda_{2}\right]}=d_{\left[\lambda_{2}\right]}[\epsilon_{H_{b}O[\lambda_{2}]}\cdot S_{a}+\epsilon_{H_{b}R[\lambda_{2}]}\cdot (1-S_{a})]\cdot \Delta [H_{b}T].$$ (10)

The ratio of Eqs. (9) and (10) is

$$\varphi \equiv \frac{{\Delta \mathrm{OD}}_{\left[\lambda_{1}\right]}}{{\Delta \mathrm{OD}}_{\left[\lambda_{2}\right]}}=D\cdot \frac{\epsilon_{H_{b}O[\lambda_{1}]}\cdot S_{a}+\epsilon_{H_{b}R[\lambda_{1}]}\cdot (1-S_{a})+\epsilon_{\mathrm{ICG}[\lambda_{1}]}\cdot C_{\text{finger}}/[H_{g}b]}{\epsilon_{H_{b}O[\lambda_{2}]}\cdot S_{a}+\epsilon_{H_{b}R[\lambda_{2}]}\cdot (1-S_{a})},$$ (11)

where $D\equiv d_{\left[\lambda_{1}\right]}/d_{\left[\lambda_{2}\right]}$. D is given by the rearrangement of Eq. (11) for the baseline time interval that spans the last 30 s prior to ICG injection ($C_{\text{finger},0}=0$ at baseline):

$$D=\varphi_{0}[\epsilon_{H_{b}O[\lambda_{2}]}\cdot S_{a,0}+\epsilon_{H_{b}R[\lambda_{2}]}\cdot (1-S_{a,0})]/[\epsilon_{H_{b}O[\lambda_{1}]}\cdot S_{a,0}+\epsilon_{H_{b}R[\lambda_{1}]}\cdot (1-S_{a,0})],$$ (12)

where $\varphi_{0}$ and $S_{a,0}$ denote the averages of φ and $S_{a}$ across the baseline interval. Note, $S_{a,0}$ is measured with a clinical pulse oximeter on the patient. Finally, with D given by Eq. (12), Eqs. (9) and (10) can be directly solved for $C_{\text{finger}}$, i.e.,

$$C_{\text{finger}}=\{\frac{\varphi }{D}\cdot [\epsilon_{H_{b}O[\lambda_{2}]}\cdot S_{a,0}+\epsilon_{H_{b}R[\lambda_{2}]}\cdot (1-S_{a,0})]-\epsilon_{H_{b}O[\lambda_{1}]}\cdot S_{a,0}-\epsilon_{H_{b}R[\lambda_{1}]}\cdot (1-S_{a,0})\}\cdot \frac{\left[H_{g}b\right]}{\epsilon_{\mathrm{ICG}[\lambda_{1}]}},$$ (13)

where the time-dependence t is implicit for $C_{\text{finger}}$ and φ, and $S_{a}$ is approximated as a constant, i.e., $S_{a,0}$, for the 4-min interval encompassing the ICG injection (see Fig. 4, mode 2) because the acute clinical pulse oximeter measurements following the ICG bolus injection are not reliable (the presence of ICG induces an artifactual drop in the clinical pulse oximeter measurement of $S_{a}$). $\left[H_{g}b\right]$ is also assumed to be constant, and its value is obtained from the closest clinical blood gas measurement to the ICG bolus injection. The $\left[H_{g}b\right]$ measurements were acquired within 12 h of the ICG bolus injections. Note, differences in $\left[H_{g}b\right]$ between the clinical blood gas measurement and ICG bolus injection timepoints are sources of errors in the calculation of $C_{\text{finger}}$.

## 7 Appendix 2: Optical Properties Measured with TR-NIRS Based on Semi-infinite Medium

*In vivo* cerebral tissue optical properties measurements at λ=785  nm ($\mu_{a}$ and $\mu_{s}^{\prime }$) were obtained with TR-NIRS. Specifically, optical properties were extracted from the fits of TR-NIRS TPSF measurements to the time-domain analytical solution of the photon diffusion equation for the semi-infinite homogeneous medium and extrapolated zero boundary condition,^76^ i.e.,

$$\phi (r,T)=0.118\phi_{1}(r,T)+0.306\phi_{2}(r,T),$$ (14)

where the solution, ϕ(r,T), is the sum of two terms that are proportional to the fluence rate, i.e., $\phi_{1}(r,T)$, and to the current or flux, i.e., $\phi_{2}(r,t)$, respectively. The proportionality coefficients, i.e., 0.118 and 0.306, are based on a refractive index n=1.4; T is the time-of-flight along the TPSF curve, and r is the SDS. For semi-infinite media

$$\phi_{1}(r,T)=\frac{c}{{\left(4\pi DcT\right)}^{3/2}}\;\exp(-\mu_{a}cT)\;\times \{\exp[-\frac{z_{0}^{2}+r^{2}}{4DcT}]-\exp[-\frac{{\left(z_{0}+2z_{b}\right)}^{2}+r^{2}}{4DcT}]\},$$ (15)

$$\phi_{2}(r,T)=\frac{1}{2}\cdot {\left(4\pi Dc\right)}^{-3/2}T^{-5/2}\;\exp(-\mu_{a}cT)\;\times [z_{0}\;\exp(-\frac{r_{1}^{2}}{4DcT})+(z_{0}+2z_{b})\exp(-\frac{r_{2}^{2}}{4DcT})]$$ (16)

where D, c, $z_{0}$, $z_{b}$, $r_{1}$, and $r_{2}$ are defined below Eq. (4).

Due to the finite width of the laser pulse and its broadening by the instrumental response of the measuring system, the measured TPSF does not represent the true impulse of the tissue. To account for this, the theoretical curve generated by Eq. (14) is convolved with the IRF (Sec. 2.3) measured with the same fiber optics.^58^ Note that $T=\tilde{T}-t_{0}$, where $\overset{˜}{T}$ is the time between the source trigger and photon detection, and $t_{0}$ is the launch time of the incident source pulse on the tissue (Sec. 2.1.1). A nonlinear optimization routine (MATLAB^®^ function fminsearchbnd) was used to obtain $\mu_{a}$, $\mu_{s}^{\prime }$, and $t_{0}$ from the fit of the measured TPSF to the convolution of the IRF and theoretical TPSF (ϕ), i.e., ${\mathrm{TPSF}}_{\text{fit}}=\mathrm{IRF}*\phi$ ($\mu_{a}$ and $\mu_{s}^{\prime }$ constrained to be between 0.001 and $1\;{\mathrm{cm}}^{-1}$, and 0.1 and $50\;{\mathrm{cm}}^{-1}$, respectively; $t_{0}$ was constrained to be within ±50  ps of the time point when the IRF maximum occurs). Fitting is the process of minimizing a cost function between measured TPSF and the ${\mathrm{TPSF}}_{\text{fit}}$, where the measured TPSF and ${\mathrm{TPSF}}_{\text{fit}}$ were normalized to the area under the curve. The cost function was defined as the sum of the squared difference between measured TPSF and the ${\mathrm{TPSF}}_{\text{fit}}$ scaled by squared root of measured TPSF at each time-of-flight. The fitting range was set to time of flights between the TPSF rising to 20% and falling to 10% of the maximal photon count.

## 8 Appendix 3: Computer Simulation to Evaluate CBF Underestimation Using Semi-Infinite Model

Computer simulation based on a two-layer model which mimics the extracerebral, i.e., scalp and skull, and cerebral tissues was performed to evaluate the underestimation in DCE-NIRS CBF measurements with the semi-infinite model. The passage of ICG bolus was simulated according to Eq. (1), where the arterial input function, $C_{a}(t)$, was obtained from a patient measurement with the ICG dose concentration at 0.2  mg/kg. Note, Eq. (1) was used twice to compute Q(t) for the extra-cerebral tissue [i.e., $Q_{ec}(t)$] and the cerebral tissue [i.e., $Q_{c}(t)$]. The impulse residue function, R(t), was modeled as an exponential, i.e., $R_{c}(t)=\exp(-t/{\mathrm{mtt}}_{c})$ and $R_{ec}(t)=\exp(-t/{\mathrm{mtt}}_{ec})$ for the cerebral and extra-cerebral tissues, respectively.^63^ Here, ${\mathrm{mtt}}_{c}$ and ${\mathrm{mtt}}_{ec}$ are the mean transit times of blood through the DCE-NIRS sampled cerebral tissue volume and extra-cerebral tissue volume, respectively. These are estimated by ${\mathrm{mtt}}_{c}=[H_{b}T_{c}]/[H_{g}b]$ and ${\mathrm{mtt}}_{ec}=[H_{b}T_{ec}]/[H_{g}b]$, where $\left[H_{b}T_{c}\right]$ and $\left[H_{b}T_{ec}\right]$ are the total hemoglobin concentrations (per volume tissue) in cerebral and extra-cerebral tissues (e.g., measured with NIRS) and $\left[H_{g}b\right]$ is the total hemoglobin concentration (per volume blood) measured from blood gas, i.e., a blood sample is drawn by the clinician in the NICU and is analyzed by a spectrophotometric measurement in the clinic labs. The ICG concentration changes in the two layers were further converted to temporal absorption changes in the two layers by multiplying the extinction coefficient, $\epsilon_{\mathrm{ICG}}$, at wavelength 808 nm according to Eq. (3). Temporal TPSF curves at certain SDS were generated with a two-layer forward solver (provided by Liemert and Kienle^77^) based on these temporal absorption changes at different layers and on constant cerebral and extra-cerebral tissue reduced scattering coefficients, tissue water content, and extra-cerebral layer thickness. Following the same method used to analyze the experiment data, the semi-infinite model was used to extract the absorption change based on the simulated TPSF curves according to Eq. (2), which was further converted to ICG concentrations. Deconvolution algorithm was then applied to the extract the absolute CBF values. The ratio between the simulated and extracted CBF values was taken to evaluate the underestimation induced by semi-infinite homogeneous model for a wide range of input parameters used to generate the simulated TPSF curves.

**Fig. 9:**
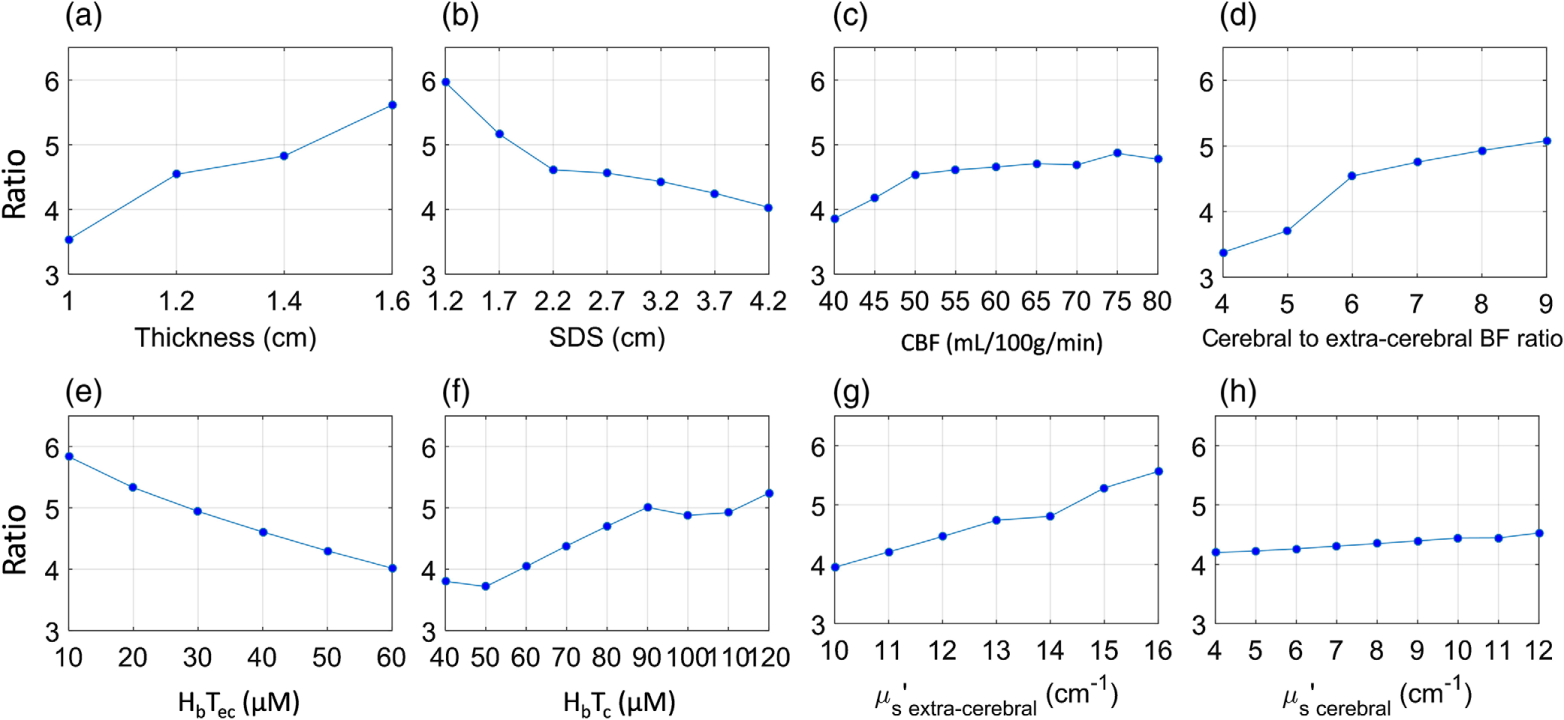
Dependence of absolute CBF underestimation with semi-infinite model (i.e., actual CBF divided by the computed CBF with the semi-infinite model) on (a) extracerebral layer thickness, (b) source–detector separation for TR-NIRS measurement, (c) CBF, (d) cerebral to extra-CBF, (e) and (f) extracerebral and cerebral total hemoglobin concentration, and (g) and (h) extracerebral and cerebral tissue reduced scattering coefficients.

Specifically, the input parameters for a “baseline model”^62^^,^^63^^,^^78^ for computer simulation are set as shown in Table 1 with SDS at 3.2 cm. Then, the input parameters were varied separately from the baseline model while all other input parameters were held constant (note, the baseline cerebral to extra-cerebral flow ratio of 6 was held constant instead of the absolute baseline extra-CBF). Variations in extra-cerebral layer thickness (from 1 to 1.6 cm), SDS (from 1.2 to 4.2 cm), CBF value (from 40 to 80  mL/100  g/min), cerebral to extra-CBF ratio (from 4 to 9 via changing extra CBF), total hemoglobin concentration in brain tissue (from 40 to 120  μM), total hemoglobin concentration in extra-cerebral tissues (from 10 to 60  μM), $\mu_{s}^{\prime }$ in extra-cerebral tissue (from 10 to $16\;{\mathrm{cm}}^{-1}$),^78^ and $\mu_{s}^{\prime }$ in cerebral tissue (from 4 to $12\;{\mathrm{cm}}^{-1}$) were tested. Figure 9 shows the dependence of the underestimation on different physiological, anatomical, and optical parameters as well as SDS. The range of underestimation varies from 3.5- to 6.0-fold, with extracerebral layer thickness being the most sensitive parameter.

**Table 1:** Baseline physiological, anatomical, and optical parameters for the forward analytical solver.

	Physiology					Optical properties (808 nm)		Anatomy
	$\left[H_{b}T\right]$ (μM)	${\mathrm{SO}}_{2}$ (%)	$\left[H_{g}b\right]$ (g/dL)	BF (mL/100 g/min)	Water Content (%)	$\mu_{s}^{\prime }$ (${\mathrm{cm}}^{-1}$)	$\mu_{a}$ (${\mathrm{cm}}^{-1}$)	Layer Thickness (cm)
Extracerebral	42	65	10	10	75	12	0.09	1.2
Cerebral	75	65	10	60	75	12	0.16	Infinite
